# Transcranial Direct Current Stimulation Reduces Negative Affect but Not Cigarette Craving in Overnight Abstinent Smokers

**DOI:** 10.3389/fpsyt.2013.00112

**Published:** 2013-09-20

**Authors:** Jiansong Xu, Felipe Fregni, Arthur L. Brody, Ardeshir S. Rahman

**Affiliations:** ^1^Department of Psychiatry, Yale Medical School, New Haven, CT, USA; ^2^Laboratory of Neuromodulation, Department of Physical Medicine & Rehabilitation, Spaulding Rehabilitation Hospital & Massachusetts General Hospital, Boston, MA, USA; ^3^Department of Psychiatry & Biobehavioral Sciences, UCLA School of Medicine, Los Angeles, CA, USA

**Keywords:** tobacco dependence, transcranial direct current stimulation, smoking abstinence, cigarette craving, negative affect, brain stimulation

## Abstract

Transcranial direct current stimulation (tDCS) can enhance cognitive control functions including attention and top-down regulation over negative affect and substance craving in both healthy and clinical populations, including early abstinent (∼1.5 h) smokers. The aim of this study was to assess whether tDCS modulates negative affect, cigarette craving, and attention of overnight abstinent tobacco dependent smokers. In this study, 24 smokers received a real and a sham session of tDCS after overnight abstinence from smoking on two different days. We applied anode to the left dorsolateral prefrontal cortex and cathode to the right supra-orbital area for 20 min with a current of 2.0 mA. We used self-report questionnaires Profile of Mood States (POMS) to assess negative affect and Urge to Smoke (UTS) Scale to assess craving for cigarette smoking, and a computerized visual target identification task to assess attention immediately before and after each tDCS. Smokers reported significantly greater reductions in POMS scores of total mood disturbance and scores of tension–anxiety, depression–dejection, and confusion–bewilderment subscales after real relative to sham tDCS. Furthermore, this reduction in negative affect positively correlated with the level of nicotine dependence as assessed by Fagerström scale. However, reductions in cigarette craving after real vs. sham tDCS did not differ, nor were there differences in reaction time or hit rate change on the visual task. Smokers did not report significant side effects of tDCS. This study demonstrates the safety of tDCS and its promising effect in ameliorating negative affect in overnight abstinent smokers. Its efficacy in treating tobacco dependence deserves further investigation.

## Introduction

Cigarette smoking is the number one cause of preventable death in the United States (1). Most tobacco dependent smokers want to stop smoking (2). However, they often experience tobacco withdrawal symptoms including craving for smoking, negative affect, and deficits in attention after abstinence from smoking, and thus relapses are common after short durations of abstinence (3–5). Current therapies for tobacco dependence include nicotine replacement therapy, bupropion sustained release formula, and varenicline. These therapies can significantly reduce tobacco withdrawal symptoms and increase abstinence rates, but are not effective in all smokers (6–9). Therefore, new therapies for reducing tobacco withdrawal are needed to help smokers quit. This study assessed whether transcranial direct current stimulation (tDCS) could modulate mood, attention, and craving for smoking of tobacco dependent smokers who maintain abstinence overnight.

Transcranial direct current stimulation is a safe, non-invasive, and inexpensive method for modulating neuronal excitability. It modulates cortical excitability using small direct electrical currents (1 ∼ 2 mA) delivered to the scalp (10–14) via two electrodes with opposite polarities (i.e., anodal and cathodal) placed on the scalp. Anodal stimulation increases cortical excitability whereas cathodal stimulation decreases cortical excitability (12, 13, 15, 16). These effects can last up to 90 min after a single stimulation session of 13 ∼ 20 min (17–20), and can further increase after repeated stimulation (i.e., cumulative effects) (21, 22).

Several studies assessed the effects of tDCS on cue-induced craving for drug use. One study applied anodal stimulation of 2 mA to either the right or left dorsolateral prefrontal cortex (DLPFC) for 20 min. This anodal stimulation reduced cue-induced tobacco craving in early abstinent (∼1.5 h) smokers (23). In another study, daily sessions (20 min) of anodal stimulation to the left DLPFC for 5 days reduced cue-induced tobacco craving and the number of smoked cigarettes during the 5-day period (22). This study also observed the cumulative effect of tDCS, i.e., cue-induced craving decreased further after each tDCS session. Other studies reported that anodal stimulation of the DLPFC reduced substance craving of alcohol abusers, marijuana users, and healthy participants (22–28). Furthermore, tDCS has been reported to induce beneficial effects on other cognitive domains such as attention, working memory, or response inhibition in healthy participants or patients with stroke, depression, Parkinson’s disease, or alcohol dependence (29–40), and also in affective domains such as mood in patients with depression or tinnitus (36, 41–45).

Findings from aforementioned studies indicate that anodal stimulation of the DLPFC may ameliorate tobacco withdrawal by reducing cigarette craving, ameliorating negative affect, and improving cognitive function. However, only two tDCS studies on smokers have been published, and these two studies assessed smokers abstinent from smoking for a short duration (i.e., ∼1.5 h). To our knowledge, no published studies assessed the effect of tDCS on longer abstinent smokers (e.g., overnight abstinent) and across several measures related to different aspects of tobacco withdrawal. Given the potential clinical significance of assessing longer abstinent smokers and also to collect pilot data for further larger clinical trials, we designed this study to test whether anodal stimulation applied to the left DLPFC in overnight abstinent smokers would modulate their mood, attention, and craving for smoking. Based on the above-reviewed studies, we predicted that abstinent smokers would show reduced craving and negative mood, and improved performance on an attention task, after real relative to sham tDCS.

## Materials and Methods

### Participants

This study was approved by the Human Investigation Committee at Yale School of Medicine and was performed in accordance with the Declaration of Helsinki. Potential participants were recruited from communities around Yale University through flyers and ads placed on Craigslist (www.craigslist.org). All participants provided written informed consent. The inclusion criteria were good general health, age between 18 and 60 years, more than 10 cigarettes per day for at least 2 years, no current illicit drug use as indicated by negative results on urine drug screens of cocaine, methamphetamine, opiates, or benzodiazepines at all sessions, and ≤10 standard drinks of alcohol per week (one standard drink consists of one 12 oz. beer, 6 oz. of wine, or one shot (1.5 oz.) of hard liquor (80 proof). The exclusion criteria were any current medical conditions, current neuropsychiatric disorders, more than one marijuana cigarette per week, pregnancy as indicated by a positive result on the urine pregnancy test, self-report of learning disability or dyslexia, current use of psychotropic drugs, or self-report of TB or HIV positive.

During a baseline session, carbon monoxide (CO) in expired air was taken as an objective measure of recent smoking (Micro Smokerlyzer II, Bedfont Scientific Instruments), and a level >15 ppm was considered as consistent with recent smoking. Participants also completed the Fagerström Test for Nicotine Dependence (46), Urge to Smoke (UTS) Scale (47), Profile of Mood States (POMS) (48), questionnaires for smoking history and demographic information, and received training in performing a computer task testing attention. The final sample included 24 smokers (3 females), with a mean age of 45 years (range 28 ∼ 59, standard deviation, SD = 7.6), smoked an average of 16.4 cigarettes per day (range 10 ∼ 30, SD = 5.6), and a mean Fagerström score of 5.7 (range 1 ∼ 9, SD = 2.0).

### Procedure

Subsequent to baseline assessments, subjects participated in two test sessions, one for real and the other for sham tDCS, on two different days with a minimal interval of 48 h. This used a single-blind design, i.e., participants were blind to real vs. sham tDCS. The sequence of the two tDCS sessions was counterbalanced among participants. On each test day smokers reported to the laboratory in the morning around 10 a.m. after maintaining abstinence from smoking overnight (>10 h abstinence). They provided a breath sample for CO assay, which should be <10 ppm or half of the baseline measure as a confirmation of overnight abstinence. Otherwise, the study session would be stopped and rescheduled. Smokers also provided a urine sample for drug screen, and would be excluded from further study if the urine sample were positive for any drugs mentioned above.

After confirmation of overnight abstinence and no drug use, participants continued the study session by completing a set of questionnaires including UTS (47) and POMS (48). Then, they performed a computerized task testing attention. After the task, they watched cigarette smoking-related pictures and video clips to induce craving for smoking for 5 min. While viewing smoking cues, smokers were instructed to put a pack of cigarettes on the desk in front of them, hold a cigarette, and put the cigarette in their mouth, and light a lighter without lighting the cigarette. Participants received tDCS (either real or sham) after watching the smoking cues. Following the tDCS, they completed the tDCS Adverse Effects Questionnaire, performed the computerized attention task, and completed the UTS and POMS again.

### Transcranial direct current stimulation

We used a 1 × 1 Low-Intensity DC Stimulator, Model 1224-B (Soterix, LLC, New York, NY, USA), and two sponge electrodes (5 cm × 7 cm) soaked with saline to deliver tDCS. During each session, the anode was placed over the left DLPFC and the cathode was placed over the contralateral supra-orbital area. The DLPFC was localized using the international 10/20 EEG system (F3) (49). In real tDCS session, stimulation was given at 2 mA for 20 min, with gradual ramping up of the current over 30 s. For sham stimulation, current ramped up to 2 mA over the first 30 s and then ramped down to zero during another 30 s, thus giving the same initial sensation of tDCS. This procedure was regularly used to keep participants blind to the real vs. sham stimulation (23, 50–52). The sham stimulation also lasted for 20 min.

### Measures

The POMS consists of six subscales, collectively including 65 five-point items that describe mood, and these items were used to calculate a score for total mood disturbance. The range of possible scores is −32 to 200 for total mood disturbance, calculated by subtracting the score for the vigor–activity subscale from the sum of scores for the remaining five subscales (5). The possible scores for the six subscales are as follows: tension–anxiety (0–36), depression–dejection (0–60), anger–hostility (0–48), vigor–activity (0–32), fatigue–inertia (0–28), and confusion–bewilderment (0–28). For the total mood disturbance and all subscales, except for vigor–activity, a higher score indicates a more negative mood state.

The UTS was used to assess cigarette craving. It consists of following seven-point items: (1) If you could smoke freely, would you like a cigarette at this moment? (2) Do you have an urge for a cigarette right now? (3) Do you miss a cigarette? (4) I crave a cigarette right now, (5) I am going to smoke as soon as possible, (6) All I want right now is a cigarette, (7) I don’t want to smoke now, (8) I have no desire for a cigarette now, (9) Nothing would be better than smoking a cigarette right now, (10) Smoking a cigarette would not be pleasant. The highest possible score is 70, and a higher score indicates greater craving. The tDCS Side Effect Questionnaire was used to assess tDCS side effects including headache, neck pain, scalp pain, scalp burns, tingling, skin redness, sleepiness, trouble concentrating, acute mood change, and other effects (24). The severity of each side effect is indexed using a four-point system, i.e., 1-Absent, 2-Mild, 3-Moderate, and 4-Severe.

**Figure 1:**
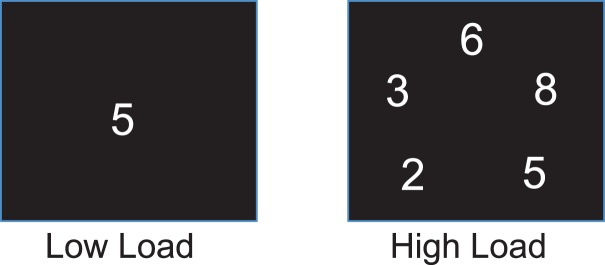
**Task stimulus**. One and five digits were presented on the screen as stimulus for the low and high load conditions, respectively.

The computerized task for testing attention had two load conditions, one for low and the other for high load. It used digits, inclusively between 1 and 9, as stimuli. The low load condition presented one digit in the center of the screen for each stimulus (Figure 1), and the stimulus was a target if the digit was an even number. The high load condition presented five digits simultaneously for each stimulus (Figure 1), and the stimulus was a target if three of the five digits were even numbers. The task used block design and each block consisted of 40 trials and one third of them were targets. Each stimulus was presented for 500 ms with an interstimulus interval of 1000 ms. Our previous studies and others’ indicated that different neural substrates underlay attention at different levels of attentional demand (53, 54). Therefore, a task with parametric loads was used to help understand whether tDCS modulates attention at specific task loads. Due to a technical problem, task performance record was not complete for four participants. Given that this missing data can be considered completely at random, the task performances of these 4 participants were excluded from analysis, and the performance data from 20 remaining participants were analyzed.

### Data analysis

SPSS paired *t*-test was used to compare scores on POMS and UTS between baseline and after overnight abstinence before tDCS. SPSS general Linear Model (GLM) for repeated measures was used to analyze the scores for POMS and UTS, and reaction time (RT) and hit rates on the attention task for assessing the effect of anodal stimulation of the left DLPFC. Because the current study assessed tobacco withdrawal symptoms of each participant repeatedly for four times in total, i.e., two times for each of two study sessions. Therefore, the difference among group means of four assessments can be tested using one-way analysis of variance (ANOVA) with repeated measures, which is implemented using GLM for repeated measures in SPSS. The scores one POMS and UTS and performance parameters were dependent variables, and test sessions and blocks (i.e., pre- vs. post-tDCS) were within-subject variables. Statistical analyses were performed to assess whether dependent variables showed significant differences in changes from before to after stimulation in real vs. sham tDCS session. SPSS binary correlation analysis was used to assess the relationship between scores on Fagerström test and changes in scores of POMS and UTS during each tDCS session. The difference in coefficients of correlations between Fagerström scores and reduction of POMS total scores after real vs. sham stimulation was assessed using a tool from a public website (55). The rates of tDCS side effects between the real and sham sessions were compared using SPSS Chi-square test. All statistical significant thresholds were set at *p* < 0.05.

## Results

### tDCS effects

**Figure 2:**
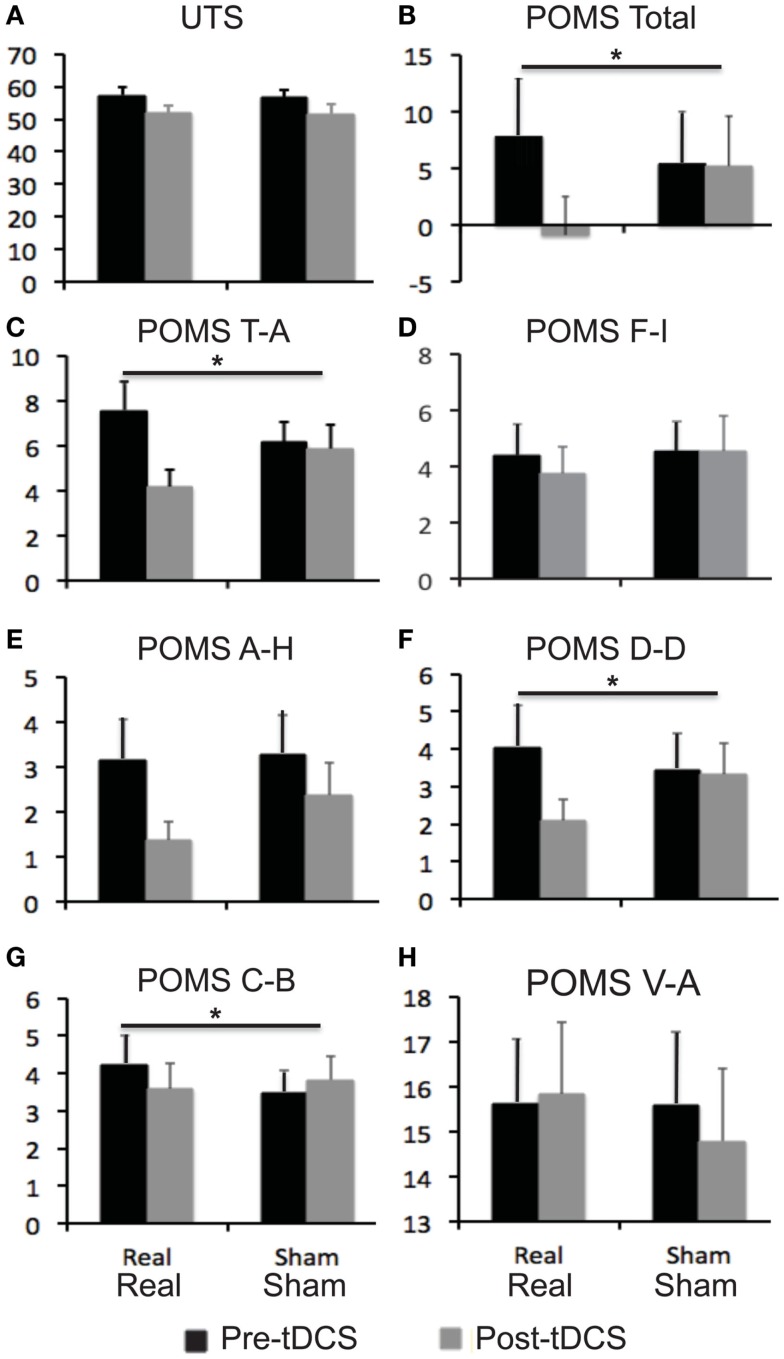
**Self-reported scores on mood and craving for smoking**. Bar graphs show self-reported scores on urge to smoke (UTS) scale and profile of mood state (POMS). **(A)** UTS scores; **(B)** POMS total scores; **(C–H)** scores for six subscales of POMS. Error bars indicate standard error of means (SE). Abbreviations: A–H, anger–hostility; C–B, confusion–bewilderment; D–D, depression–dejection; F–I, fatigue–inertia, T–A: tension–anxiety, V–A: vigor–activity.

**Figure 3:**
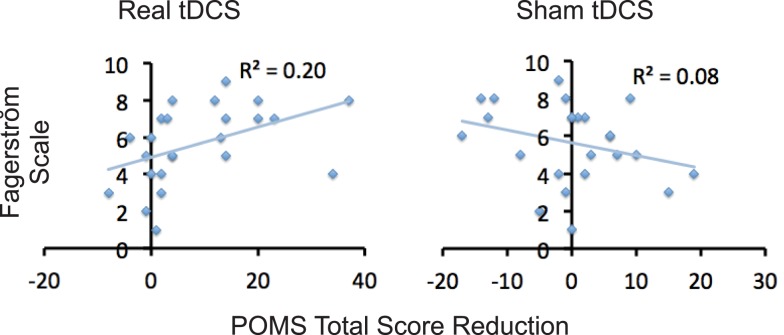
**Correlations between level of nicotine dependence and tDCS-induced reduction of negative affect**. Scatter plots demonstrate correlations between scores on Fagerström and reduction in POMS total scores after tDCS relative to before tDCS.

Relative to baseline, participants did not report significantly greater scores on POMS after overnight abstinence before tDCS on both days of real and sham stimulation (Table 1). Several POMS scores showed a main effect of block, i.e., a significant reduction after tDCS relative to before tDCS. They included the total score [*F*(1, 23) = 13.41, *p* = 0.001] (Figure 2B) and scores for subscales tension–anxiety [*F*(1, 23) = 23.36, *p* < 0.001] and anger–hostility [*F*(1, 23) = 10.25, *p* = 0.004] (Figures 2C,E). Furthermore, several POMS scores showed a significant effect of session × block two-way interaction, i.e., a greater reduction after real relative to sham tDCS. They included POMS total scale [*F*(1, 23) = 7.7, *p* = 0.011] and subscales for tension–anxiety [*F*(1, 23) = 5.1, *p* = 0.033], depression–dejection [*F*(1, 23) = 9.2, *p* = 0.006], and confusion–bewilderment [*F*(1, 23) = 4.9, *p* = 0.037] (Figures 2B,C,F,G). The Fagerström score of smokers positively correlated with the reduction of POMS total scores after real stimulation (*N* = 24, *r* = 0.451, *p* = 0.027), but not after sham stimulation (*N* = 24, *r* = −0.285, *p* = 0.176) (Figure 3). The correlations between Fagerström scores and reduction of POMS total scores after real vs. sham stimulation tend to be different significantly (*Z* = 1.76, *p* = 0.078). Abstinent smokers did not report significant tDCS-related changes in scores for subscales fatigue–inertia [*F*(1, 23) = 0.74, *p* = 0.398] and vigor–activity [*F*(1, 23) = 1.03, *p* = 0.32].

**Table 1:** **UTS and POMS scores at baseline and before tDCS at each session**.

		UTS	POMS total	T–A	A–H	F–I	D–D	C–B	V–A
Baseline		44.4 (16.2)	3.3 (18.8)	5.0 (4.4)	2.6 (3.2)	3.9 (4.0)	4.7 (5.4)	4.1 (3.0)	17.0 (5.7)
Real		57.4 (11.5)	7.8 (25.2)	7.6 (6.4)	3.2 (4.4)	4.4 (5.3)	4.0 (5.5)	4.3 (3.8)	15.6 (7.1)
Sham		56.9 (10.1)	5.4 (22.3)	6.2 (4.3)	3.3 (4.1)	4.5 (5.1)	3.5 (4.6)	3.5 (2.8)	15.6 (8.0)
Baseline vs. real	*t*	4.7	0.94	1.60	0.73	0.79	0.63	0.22	1.12
	*p*	<0.001	0.36	0.12	0.47	0.44	0.54	0.83	0.27
Baseline vs. sham	*t*	4.6	0.44	1.01	0.79	0.74	1.02	1.16	1.07
	*p*	<0.001	0.67	0.32	0.44	0.46	0.32	0.26	0.30

Number in the parenthesis indicates standard deviation (SD). Abbreviations: A–H, anger–hostility; C–B, confusion–bewilderment; D–D, depression–dejection; F–I, fatigue–inertia; T–A, tension–anxiety; V–A, vigor–activity.

Relative to baseline, participants reported a significantly greater score on UTS after overnight abstinence before tDCS on both days of real and sham stimulation (Table 1). Score on UTS showed a main effect of block [*F*(1, 23) = 13.8, *p* = 0.001], i.e., a significant reduction after tDCS relative to before tDCS (Figure 2A). However, it did not show an interaction effect of session × block [*F*(1, 23) = 0.009, *p* = 0.927] (Figure 2). The changes of UTS score after real vs. sham tDCS did not correlate with each other significantly (*N* = 24, *r* = 0.066, *p* = 0.76). Participants did not show significant correlations between Fagerström scores and changes on UTS after real (*N* = 24, *r* = 0.23, *p* = 0.279) or sham (*N* = 24, *r* = −0.152, *p* = 0.479) stimulation, nor between changes on scores of UTS and POMS after real (*N* = 24, *r* = 0.251, *p* = 0.237) or sham (*N* = 24, *r* = 0.330, *p* = 0.115) stimulation.

**Figure 4:**
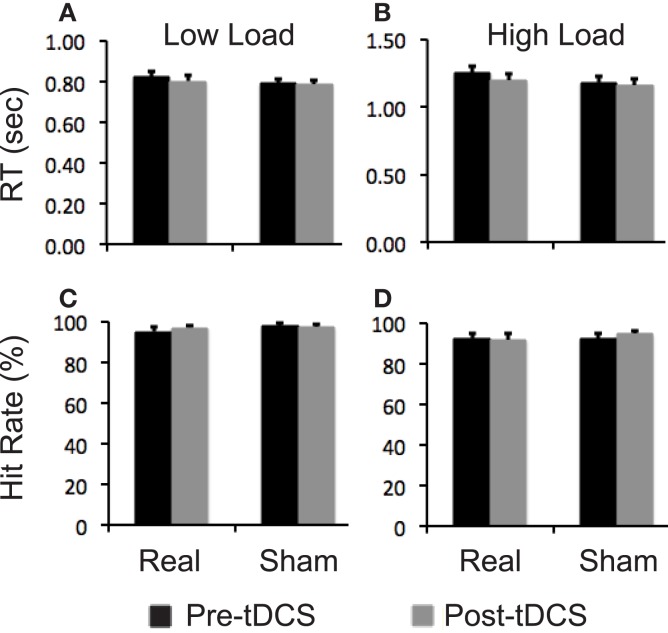
**Task performance data**. Bar graphs show reaction time (RT) and hit rates on the visual attention task. **(A,B)** RT at low and high task load condition, respectively; **(C,D)** hit rates at low and high task load condition, respectively. Error bars indicate standard error of means (SEs).

Participants did not show a significantly different change in any performance measures of the attention task at either task load after real relative to sham stimulation (Figure 4).

### tDCS side effects

The most commonly reported side effects were tingling, sleepiness, and scalp burn (Table 2). These side effects were usually mild and did not prevent any participants from completing tDCS (Table 2). Though more participants reported tingling and sleepiness in the real relative to sham session, this difference between the two sessions did not reach statistical significance, indicating that the blinding method was effective for the current participants.

**Table 2:** **Number of participants reported tDCS side effects**.

tDCS session	Headache	Neck pain	Scalp pain	Scalp burns	Tingling	Skin redness	Sleepiness	Trouble concentrating	Acute mood change
Real (severity)	0	0	1 (2.0)	4 (2.3)	19 (2.1)	0	7 (2.6)	1 (2.0)	1 (3.0)
Sham (severity)	2 (2.0)	1 (2.0)	1 (2.0)	4 (2)	13 (2.1)	1 (2.0)	5 (2.6)	1 (3.0)	1 (2.0)
*X*^2^ value	2.18	1.07	0.001	0.004	2.77	1.07	0.341	0.001	0.001
*p*	0.140	0.302	0.975	0.947	0.096	0.301	0.559	0.975	0.975

Number in the parenthesis indicates group mean of severity of reported side effect.

## Discussion

To our knowledge, this study is the first to assess the effect of tDCS on mood, attention, and craving for smoking of tobacco dependent smokers after overnight abstinence. The main finding was that anodal stimulation to the left DLPFC reduced negative affect of overnight abstinent smokers. This reduction in negative affect positively correlated with the level of nicotine dependence as measured by the Fagerström test. However, tDCS did not show significant effect on cigarette craving or performance on a visual attentional task.

### Negative affect

Chronic smoking may impair structure and function of the brain including the DLPFC. For example, chronic smoking desensitizes nicotinic acetylcholinergic receptors and increases their density in the brain including the DLPFC (56–59), reduce gray matter density in the DLPFC (60, 61), and alter task-related activity of the DLPFC during an attentional task (62). Overnight abstinence from smoking may further alter functional activity of the DLPFC and other brain regions in tobacco dependent smokers and leads to withdrawal symptoms (63–67). The smokers in the current study reported an increased total score of POMS after overnight abstinence relative to baseline, though this increase did not reach the threshold of statistical significance. The short duration of abstinence (∼10 h) of current study may contribute to this non-significant increase in negative affect, because tobacco dependent smokers often reported a significant increase in negative affect a longer duration (∼24 h) of abstinence (68, 69).

The functional activity in the left and right DLPFC is associated with positive and negative affect, respectively (70–72). Clinical depression is associated with reduced activity in the left DLPFC and increased activity in the right DLPFC (73). Anodal stimulation can reduce intracortical inhibition (74), and increase the functional activity of stimulated cortex (75–78). Several studies find that anodal stimulation of the left DLPFC reduces depressive symptoms of patients with major depression (36, 37, 41, 79), improves mood of patients with chronic tinnitus (80), and decreases negative ratings of pictures with negative valence in healthy participants (81). In the current study, real anodal stimulation of the left DLPFC significantly reduced negative affect of overnight abstinent smokers, and this effect positively correlated with the level of nicotine dependence, suggesting that tDCS is especially effective in heavy smokers. Based on above-reviewed literature, we predict that this effect of tDCS on negative affect is mediated by increased activity in the left DLPFC after anodal stimulation. This prediction can be tested in future studies using fMRI.

### Craving for smoking

Different from no significant changes in negative affect after overnight abstinence, smokers reported a significant increase in craving for smoking. Real anodal stimulation of the left DLPFC did not significantly reduce craving relative to sham stimulation. This data is also different from the significant reduction in negative affect after real stimulation. Therefore, both the overnight abstinence and real anodal stimulation to the left DLPFC showed dissociable effects on negative affect vs. craving for smoking, indicating that the two common tobacco withdrawal symptoms have different neural mechanisms.

The current negative finding of tDCS on craving for smoking is different from previous findings of reduction in cue-induced craving after real tDCS (22, 23). A major difference between the current and the two previous tDCS studies is that the current study assessed overnight abstinent smokers while the previous studies assessed minimally abstinent (∼1.5 h) smokers. A recent study reported that overnight abstinence from smoking reduced the excitability increase of the primary motor cortex induced by anodal stimulation in tobacco dependent smokers, and that nicotine administration to overnight abstinent smokers reestablished the excitability increase of the motor cortex induced by anodal stimulation (82). Therefore, overnight abstinence from cigarette smoking may reduce the enhancing effect of anodal stimulation on cortical excitability in tobacco dependent smokers. This effect of overnight abstinence might contribute to the current negative finding on smoking craving after anodal stimulation of the left DLPFC. In addition, other factors, such as different participants, study procedures, and craving measures might also contribute to this different finding between current and previous studies.

### Cognitive function

No published studies have assessed the effect of tDCS on cognitive function of abstinent smokers. However, multiple tDCS studies report that anodal stimulation of the left DLPFC improves cognitive function including attention and working memory of healthy participants or patients with depression (29, 38, 40, 83, 84). In the current study, anodal stimulation of the left DLPFC did not improve the performance of abstinent smokers on an attentional task. This negative finding may due to the specific task used in the current study, or other factors related to the negative finding on craving for smoking as discussed in the above section.

### tDCS side effect

Consistent with previous tDCS studies, participants did not report serious side effects of tDCS in the current study. More participants reported tingling and sleepiness after real relative to sham stimulation. However, this difference between real and sham stimulation did not reach statistical significance.

### Limitations

The main limitation of the current study is a small sample size and a short duration of abstinence. Tobacco withdrawal may starts within a few hours of abstinence and will reach peak around 24 h after abstinence (68). Therefore, the effect of tDCS on negative affect should be assessed again when negative affect reaches its peak (e.g., after 24 h abstinence). Another limitation is no debriefing after the second tDCS, and therefore it is not clear whether the participants are really blind to the stimulation conditions.

In summary, tDCS is a safe, inexpensive, and easy to use method for modulating cortical excitability. Anodal stimulation of the left DLPFC showed a promising effect on negative affect of overnight abstinent smokers. Future studies should explore approaches, such as concurrent nicotine administration and behavioral therapy, or repeated sessions, for further enhancing the efficacy of anodal stimulation in ameliorating tobacco withdrawal in abstinent smokers.

## Conflict of Interest Statement

The authors declare that the research was conducted in the absence of any commercial or financial relationships that could be construed as a potential conflict of interest.
